# Biological Activity and Phytochemical Characteristics of Star Anise (*Illicium verum*) Essential Oil and Its Anti-*Salmonella* Activity on Sous Vide Pumpkin Model

**DOI:** 10.3390/foods13101505

**Published:** 2024-05-13

**Authors:** Miroslava Kačániová, Nenad L. Vukovic, Natália Čmiková, Alessandro Bianchi, Stefania Garzoli, Rania Ben Saad, Anis Ben Hsouna, Joel Horacio Elizondo-Luévano, Hussein A. H. Said-Al Ahl, Wafaa M. Hikal, Milena D. Vukic

**Affiliations:** 1 Institute of Horticulture, Faculty of Horticulture and Landscape Engineering, Slovak University of Agriculture, Tr. A. Hlinku 2, 94976 Nitra, Slovakia; n.cmikova@gmail.com (N.Č.); milena.vukic@pmf.kg.ac.rs (M.D.V.); 2 School of Medical & Health Sciences, University of Economics and Human Sciences in Warsaw, Okopowa 59, 01 043 Warszawa, Poland; 3 Department of Chemistry, Faculty of Science, University of Kragujevac, 34000 Kragujevac, Serbia; nvchem@yahoo.com; 4 Department of Agriculture, Food and Environment, University of Pisa, Via del Borghetto 80, 56124 Pisa, Italy; alessandro.bianchi@phd.unipi.it; 5 Department of Chemistry and Technologies of Drug, Sapienza University, P. le Aldo Moro, 5, 00185 Rome, Italy; stefania.garzoli@uniroma1.it; 6 Laboratory of Biotechnology and Plant Improvement, Centre of Biotechnology of Sfax, B.P “1177”, Sfax 3018, Tunisia; raniabensaad@gmail.com (R.B.S.); benhsounanis@gmail.com (A.B.H.); 7 Department of Environmental Sciences and Nutrition, Higher Institute of Applied Sciences and Technology of Mahdia, University of Monastir, Monastir 5000, Tunisia; 8 Faculty of Agronomy, Universidad Autónoma de Nuevo León (UANL), Av. Francisco Villa S/N, Col. Ex Hacienda el Canadá, General Escobedo 66050, Nuevo León, Mexico; joel.elizondolv@uanl.edu.mx; 9 Medicinal and Aromatic Plants Research Department, Pharmaceutical and Drug Industries Research Institute, National Research Centre (NRC), 33 El-Behouth St. Dokki, Giza 12622, Egypt; shussein272@yahoo.com; 10 Department of Biology, Faculty of Science, University of Tabuk, Tabuk 71491, Saudi Arabia; wafaahikal@gmail.com

**Keywords:** phytochemical composition, star anise, *in situ* antimicrobial effect on pear and beetroot, *in vitro* activity against G^+^ and G^−^, anti-*Salmonella* effect on pumpkin model

## Abstract

*Illicium verum*, commonly known as star anise, represents one of the notable botanical species and is recognized for its rich reservoir of diverse bioactive compounds. Beyond its culinary application as a spice, this plant has been extensively utilized in traditional medicine. Given the contemporary emphasis on incorporating natural resources into food production, particularly essential oils, to enhance sensory attributes and extend shelf life, our study seeks to elucidate the chemical composition and evaluate the antibacterial (*in vitro*, *in situ*) and insecticidal properties of *Illicium verum* essential oil (IVEO). Also, microbiological analyses of pumpkin sous vide treated with IVEO after inoculation of *Salmonella enterica* were evaluated after 1 and 7 days of study. GC/MS analysis revealed a significantly high amount of (*E*)-anethole (88.4%) in the investigated EO. The disc diffusion method shows that the antibacterial activity of the IVEO ranged from 5.33 (*Streptococcus constellatus*) to 10.33 mm (*Citrobacter freundii*). The lowest minimal inhibition concentration was found against *E. coli* and the minimum biofilm inhibition concertation was found against *S. enterica*. In the vapor phase, the best antimicrobial activity was found against *E. coli* in the pears model and against *S. sonei* in the beetroot model. The application of the sous vide method in combination with IVEO application decreased the number of microbial counts and eliminated the growth of *S. enterica*. The most isolated microbiota identified from the sous vide pumpkin were *Bacillus amyloliquefaciens*, *B. cereus*, *B. licheniformis*, and *Ralstonia picketii*. Modifications to the protein composition of biofilm-forming bacteria *S. enterica* were suggested by the MALDI TOF MS instigations. The IVEO showed insecticidal potential against *Harmonia axyridis*. Thanks to the properties of IVEO, our results suggest it can be used in the food industry as a natural supplement to extend the shelf life of foods and as a natural insecticide.

## 1. Introduction

To address the issue of food spoilage and combat harmful bacteria, the food industry is increasingly turning to natural compounds as new solutions for food preservation, all while fostering innovations in packaging [1]. Preservatives used by manufacturers mainly include chemicals with antioxidant and antiseptic properties that inhibit the growth of mold and bacteria [2]. The improper usage of antibiotics represents a significant risk to public health, highlighted by the rise in bacterial resistance. The food chain and the environment serve as primary pathways for the development of antibiotic resistance in humans, facilitating the transfer of resistant bacteria to individuals [3]. The usage of synthetic antioxidant molecules is also linked to potential toxicological hazards [4]. As a result, the research into natural food preservatives has been prompted last few decades [5]. The greatest attention in this field has been given to plants and their products. Concerning this issue, the main advantage of medicinal plants is that they are a dependable source of bioactive chemicals that have been recognized for their therapeutic qualities [6]. As representatives of plant products, essential oils (EOs) are currently being utilized as natural preservatives. With antibacterial properties akin to chemical preservatives, their distinct advantage lies in their safety, non-toxicity, and environmental friendliness. The escalating adoption of EOs as replacements for chemical preservatives in the food industry stems from their superior efficacy and rising acceptance within the human body [7].

Raw or partially cooked foods can undergo sous vide preparation, a method involving sealing the foods in a plastic pouch and slow cooking them in a water bath at temperatures between 65 °C and 95 °C for an extended duration [8]. To prevent health risks, immediate freezing is essential if the sous vide product is intended for later use [9]. The practice of storing sous vide food in vacuum-sealed pouches is a standard procedure aimed at reducing cross contamination. Safety concerns primarily revolve around the potential toxicity of pathogenic microorganisms that can survive the gentle heat treatments used during refrigeration [10]. Sous vide cooking does not require any additional additives such as preservatives [11]. Combining EOs with vegetables with strong flavorings could improve the taste of the final product and ensure microbiological quality [12]. The research findings demonstrated that combining EOs with vacuum sealing, alongside chilled storage, effectively inhibited the growth of mesophilic bacteria and Enterobacteriaceae in minimally processed potatoes designated for sous vide cooking, even after an 11-day storage period [13].

One of the most common foodborne pathogens, Gram-negative *Salmonella enterica*, represents the third leading cause of human death due to diarrheal infections worldwide [14]. In the European Union (EU) salmonellosis (an infection caused by *Salmonella*) is ranked as the second most reported gastrointestinal condition [15]. According to the Centers for Disease Control and Prevention (CDC), approximately 95% of all infections caused by this bacterium are attributed to foodborne sources [16]. When compared to other pathogens, *Salmonella* has been associated with several outbreaks [15]. One such outbreak, associated with contaminated eggs, led to over 1500 reported cases of salmonellosis across Slovakia, Poland, and Spain [15]. Even though *Salmonella* has mostly been associated with egg products, new investigations show that it can also be found in some fresh food (baby spinach, tomatoes, lettuce, peppers, basil, etc.). Moreover, the microorganism’s capacity to create biofilms enhances its ability to survive on food items [17]. Currently, methods including food processing and thermal treatments are considered to be the most effective for elimination of this highly virulent microorganism [18]. Nonetheless, the development of new intervention protocols and techniques is needed to reduce the possibility of produce being contaminated by this pathogen.

Apart from its widespread usage in China, star anise fruit occupies a significant role in traditional Indian medicine, being utilized to alleviate diverse conditions including cough, asthma, rheumatism, facial paralysis, dyspepsia, flatulence, diarrhea, and spasmodic colonalgia. The essential oil derived from this fruit is deemed safe by the FDA and has demonstrated therapeutic properties in treating various ailments such as cramping pain, flatulence, spasms, and rheumatism [19]. Additionally, studies show that *Illicium verum*, or star anise EO, is characterized by diverse biological properties such as antimicrobial, antioxidant, insecticidal, and anti-inflammatory [20]. Star anise EO contains compounds belonging to different categories including phenylpropanoids, monoterpene hydrocarbons, sesquiterpene hydrocarbons, and their oxygenated derivatives [20]. The primary component of star anise EO is anethole, responsible for its characteristic flavor. Additionally, this phenylpropanoid is known for its antiparasitic, antiviral, antibacterial, and antifungal properties [21]. Additionally, prior screening for novel agrochemicals from Chinese medicinal herbs demonstrated the insecticidal activity of *Illicium verum* essential oil (IVEO) against *Sitophilus zeamais* and *Cryptolestes pusillus* Schönherr [22]. Subsequent research on IVEO revealed its fumigant and repellent properties against *Blattella germanica*, *S. zeamais*, *Lasioderma serricorne*, *Sitophilus oryzae*, *Callosobruchus chinensis*, and *Aedes aegypti* [23,24,25].

Prompted by the emergency of the presented issues, and the proven beneficial effects of IVEO, this study aimed to assess the chemical composition of this EO and explore its antibacterial (*in vitro* and *in situ*), antibiofilm, and insecticidal activities. Additionally, the investigation sought to contribute valuable data to support the potential use and development of IVEO as a green storage protectant, specifically in the control of stored products such as sous vide pumpkin inoculated with the pathogenic bacteria *Salmonella enterica*.

## 2. Materials and Methods

### 2.1. Essential Oil

The essential oil (EO) utilized in this study was obtained through distillation of dried fruits of *Illicium verum* and was provided by Hanus s.r.o. (Nitra, Slovakia). The fruits employed for the extraction of the EO were sourced from China and stored at 4 °C in darkness prior to analysis.

### 2.2. GC and GC/MS Chemical Analyses of IVEO Sample

The chemical composition of *Illicum verum* EO was analyzed using a 6890 N gas chromatograph coupled with a quadrupole mass spectrometer 5975 B (Agilent Technologies, Santa Clara, CA, USA). Additionally, the semi-quantitative percentage amounts of each identified compound were determined using a 6890 N gas chromatograph coupled with an FID detector (Agilent Technologies, Santa Clara, CA, USA). HP Enhanced ChemStation software D.03.00.611 (Agilent Technologies, Santa Clara, CA, USA) was used for the acquisition and interpretation of mass spectra and chromatographic data [26]. In order to separate volatile constituents from the complex mixture of analysis essential oil sample, an HP-5MS capillary column ((5%-phenyl) methylpolysiloxane; 30 m length; 0.25 mm internal diameter; 0.25 µm film thickness) was installed in GC/MS and GC ovens. The injection volume of the 10% solution of essential oil in hexane was set at 1 µL, while helium 5.0 was used as a carrier gas with a flow rate of 1 mL/min. The temperatures of the split/splitless injector, MS source, and MS quadruple were kept at 280 °C, 230 °C, and 150 °C, respectively. The split ratio was adjusted to 40.8:1. Mass spectra data acquisition was conducted within the mass scan range of 35–550 amu, with an ionization energy of 70 eV. The oven temperature was programmed as follows: starting from 50 °C, it increased to 75 °C at a rate of 3 °C/min and was held for 4 min; then, it was raised from 75 °C to 120 °C at a rate of 5 °C/min and held for 2 min; finally, it increased from 120 °C to 290 °C at a rate of 5 °C/min. The total run time for the analysis was 57.33 min. Identification of individual volatile constituents was performed by comparing their mass spectra with those stored in the MS library (Wiley/Nist) and through a comparison of the retention indices (RI) of compounds identified in the sample with those of series n alkanes (C_7_–C_35_). The percentages of the identified compounds (amounts higher than 0.1%) were derived from their GC peak areas [27,28].

### 2.3. Microbial Strains for Antimicrobial Activity

The assessed EO antibacterial efficacy was evaluated using the following strains of bacteria: Gram-negative bacteria (G^−^) included *Citrobacter freundii* CCM 7187, *Escherichia coli* CCM 3954, *Serratia marcescens* CCM 8587, *Shigella sonnei* CCM 4421. Gram-positive bacteria (G^+^) included *Priestia* (*Bacillus*) *megaterium* CCM 2007, *Enterococcus faecalis* CCM4224, *Streptococcus constellatus* CCM 4043, *Streptococcus pneumoniae* CCM 4501. All G^+^ and G^−^ bacterial species were obtained from the Czech Collection of Microorganisms (CCM), which is housed in Brno, Czech Republic. To evaluate the antibiofilm efficacy, biofilm-forming G^−^ *Salmonella enterica* strains were isolated and sequenced from milk production. The bacterial inoculum was cultivated in Mueller–Hinton Broth (MHB, Oxoid, Basingstoke, UK) for 24 h at 37 °C before analysis. On the day of the experiment, the optical density of the bacterial inoculum was adjusted to 0.5 using the McFarland standard.

### 2.4. Disc Diffusion Method

The disk diffusion susceptibility test was conducted using the aforementioned microbial strains. Mueller–Hinton Agar (MHA) was inoculated with 100 µL of bacterial strains cultured in Mueller–Hinton Broth (MHB). Circular discs, saturated with 10 µL of the tested IVEO, were then placed onto the surface of the agar. Following an incubation period of 24 h at 37 °C, the inhibitory zones were measured and recorded in millimeters. Cefoxitin and gentamicin antibiotics (30 µg/disc, Oxoid, Basingstoke, UK) served as positive controls for G^+^ and G^−^ microorganisms, respectively. The entire experiment was performed in triplicate to ensure precision and reproducibility [29].

### 2.5. Broth Dilution Method

The calculation of minimum inhibitory concentration (MIC) values, specifically MIC_50_ and MIC_90_, followed established procedures outlined by Kačániová et al. [30]. In summary, 50 μL of microbial inoculum was dispensed into each well of a 96-well microtiter plate. Various concentrations of IVEO were added to Mueller–Hinton Broth (MHB) within the range of 10 mg/mL to 0.00488 mg/mL. Positive control wells contained MHB with inoculum to ensure maximal growth, while negative control wells contained MHB with IVEO at the designated concentration. Following incubation, absorbance readings at 570 nm were taken using a spectrophotometer (Glomax, Promega Inc., Madison, WI, USA). The MIC_50_ represented the lowest concentration of EO inhibiting 50% of bacterial growth, whereas the MIC_90_ denoted the lowest concentration inhibiting 90% of bacterial growth. The entire experiment was performed in triplicate to ensure consistency and reliability [29].

### 2.6. Vapor Phase of IVEO

IVEO vapor phase antibacterial activity was assessed against specific yeast and bacterial strains described in Section 2.3. using pears and beetroots as substrates. The experimental procedure mirrored a previous study [31], with both the fruit and vegetable being cut into 0.5 mm pieces, dried, and cleaned before bacteria were introduced to Petri plates with agar. IVEO, dissolved in 99.8% ethyl acetate (Sigma-Aldrich, St Louis, MO, USA) at varying concentrations (500, 250, 125, and 62.5 mg/L), was applied to sterile filter paper, while control sheets were exposed only to ethyl acetate. The Petri dishes were sealed, and after a minute for ethyl acetate evaporation, they were incubated at 37 °C for seven days. Bacterial growth was measured *in situ* using both traditional methods and ImageJ software 1.8.0. The volume density of bacterial colonies (vv) was calculated, and the percentage of bacterial growth inhibition (BGI) resulting from the EO vapor phase treatment was determined using the provided formulas [31].

(1) $$vv\;(\%)=\frac{P}{p}$$

where P is the stereological grid points that strike the colonies and p is the points that fall inside the reference space (growth substrate used).

The EO vapor phase treatment resulted in a percentage (%) of bacterial growth inhibition (BGI), which was calculated as follows:

(2) $$BGI=\frac{C-T}{C}\times 100$$

where C and T represent the control and treatment groups, respectively. Each group represents bacterial growth expressed as *v/v* and results obtained as negative values indicate growth stimulation.

### 2.7. Sous Vide Antimicrobial Affect

For this study, pumpkin samples were procured from an authorized dealer in the Slovak Republic, totaling 2.5 kg of cleaned pumpkin. After refrigeration, the pumpkins were transported to the microbiology laboratory and subsequently cut into 5 g pieces using a sterile knife. The knife was sterilized with ethanol after each slicing. A total of 480 five-gram samples were prepared, comprising three raw samples, 240 treated and control samples on day 1, and 240 treated and control samples on day 7. Following treatment with a 100 µL solution of 1% v/w IVEO dissolved in rapeseed oil, the chopped pumpkin samples weighing 5 g each were individually vacuum-packed in polyethylene bags using a Concept vacuum packer. Control samples were prepared as both vacuum-packed and unpackaged samples. To simulate the presence of *S. enterica*, samples containing 100 µL of *S. enterica* (0.5 McFarland) and 1% v/w IVEO were prepared without damaging the pumpkin. The samples were then vacuum-packed after stirring for about a minute [32]. For the experiment, *Salmonella enterica* was utilized. Muller–Hinton agar (MHA, Oxoid, Basingstoke, UK) was used to culture the microbial inoculum for a duration of 24 h at 37 °C. After adjusting the inoculum to an optical density of 0.5 McFarland standard, or 1.5 × 10^8^ CFU/mL, 100 μL was added to the samples of pumpkin.

We had access to the following information while we were studying it:

(i) Control: Fresh pumpkin samples were treated for five to twenty-five minutes at 50 to 65 °C after being in polyethylene bags and kept at 4 °C.
(ii) Control + vacuum: Fresh pumpkin samples were treated by heating for five to twenty-five minutes at 50–65 °C in water bath after being vacuum-packed in polyethylene bags and kept at 4 °C.
(iii) EO: Fresh pumpkins that had been vacuum-packed and treated with 1% IVEO were kept at 4 °C and then heated in water bath for five to twenty-five minutes at 50 to 65 °C.
(iv) *Salmonella*: Fresh pumpkins treated with *S. enterica* and vacuum-packed were kept at 4 °C before being exposed to the bacteria and then heated in a water for five to twenty-five minutes at 50 to 65 °C.
(v) *Salmonella* + EO: Vacuum-packed fresh pumpkins treated with *S. enterica* and containing 1% IVEO were kept at 4 °C before being treated and were then heated at 50 to 65 °C for five to twenty-five minutes in water bath.

On the initial day of the experiment, raw, uncooked pumpkins were utilized to create the control samples. Following the application, gentle mixing, and blending of the EO from the first set of samples and the *S. enterica* from the second group of samples, all samples were macerated for a full day. The samples were then prepared using the CASO SV1000 sous vide equipment, which was manufactured by a company headquartered in Arnsberg, Germany. Subsequently, the prepared samples were stored for 24 h prior to cooking and then stored for an additional 7 days.

In order to prepare the samples for sous vide cooking, they were separated into groups and cooked at a specified temperature and for a specified duration under careful monitoring. The high-barrier polyethylene vacuum packaging bags consist of an impermeable material ranging between 40 and 200 microns in thickness, effectively shielding the contents from moisture as well as extreme temperatures ranging from −30 to +100 °C. The accompanying data sheet asserts their exceptional longevity, ensuring a prolonged shelf life while maintaining the quality of the enclosed food items, including taste and aroma. Furthermore, they are guaranteed to be free from plasticizers such as bisphenol A and microplastics, rendering them safe for consumption. These bags are designed to maintain their integrity for several years when stored in freezers or refrigerators.

### 2.8. Antibiofilm Assay

#### 2.8.1. Crystal Violet Study

A study by Kačániová et al. [30] investigated minimal biofilm inhibitory concentration (MBIC). Bacterial suspensions were cultured in Mueller–Hinton Broth (MHB) at 37 °C in an aerobic environment. An inoculum with an optical density of 0.5 McFarland standard was created. A 96-well microtiter plate was utilized, into which 50 μL of the inoculum and 100 μL of Mueller–Hinton Broth (MHB) were added. The first column received 100 μL of IVEO, resulting in a two-fold dilution ranging from 100 mg/mL to 0.049 mg/mL. The negative control consisted of MHB with EO, while the positive control for maximal growth included MHB with a bacterial inoculum. Subsequently, the wells were allowed to dry before being stained with 200 μL of 0.1% w/v crystal violet. After rinsing, the staining was reconstituted with 200 μL of 33% acetic acid. Using the Glomax spectrophotometer, absorbance at 570 nm was measured. MBIC, representing the concentration inhibiting biofilm formation, was determined, with MBIC_50_ and MBIC_90_ denoting the lowest doses inhibiting 50% and 90% of biofilm formation, respectively.

#### 2.8.2. MALDI-TOF MS Biotyper

Protein degradation of biofilm growth was assessed following the methodology from a previous study [30] using the Bruker Daltonics MALDI-TOF MicroFlex instrument. *S. enterica* biofilm-forming inoculum and MHB were introduced into polypropylene tubes containing glass and stainless-steel slides. IVEO was incorporated, and the tubes were then incubated for a period ranging from 3 to 14 days. Each day, biofilms were extracted, and planktonic cells from control samples devoid of EO were examined. MALDI-TOF spectra were obtained for both pellets and swabs, generating dendrograms utilizing 19 standard global spectra (MSP).

### 2.9. Insecticidal Activity

To assess IVEO’s insecticidal effectiveness, *Harmonia axyridis* (imago) served as the model organism. Following the methodology from a previous study [33], sterile filter paper in Petri plates was treated with varying concentrations of IVEO—100, 50, 25, 12.5, 6.25, and 3.125%. Each plate contained 100 individuals. The control group was treated with 0.1% polysorbate 80 (Sigma-Aldrich, St Louis, MO, USA). After 24 h, the populations of live and dead individuals were enumerated in three independent analyses.

### 2.10. Statistical Analysis

The experimental assessments were conducted in triplicate, and the results are presented as mean values with corresponding standard deviations (SD). Statistical analyses, including one-way ANOVA and Tukey’s HSD test at a significance level of *p* < 0.05, were carried out using CoStat version 6.451 (CoHort Software, Pacific Grove, CA, USA). Graphical representations were generated using JMP Pro 17.0 software package from SAS Institute, Cary, NC, USA.

## 3. Results

### 3.1. GC/MS Analysis

The IVEO volatile oil’s chemical composition was analyzed through the GC/MS technique, and the findings are outlined in Table 1 where the percentage distribution of identified compounds across various classes is shown. The analysis identified 15 compounds, constituting 97.2% of the total oil composition. The results indicate that the tested oil is predominantly characterized by a substantial amount of phenylpropanoids, notably featuring a high proportion (88.4%) of (*E*)-anethole.

### 3.2. Disc Diffusion Method

Table 2 presents the antimicrobial activity of IVEO. The most effective antimicrobial activity against G^+^ bacteria was observed with *E. faecalis* (7.33 mm), followed by *S. pneumoniae* (6.33 mm) and *B. megatherium* (5.67 mm). All the tested G^+^ bacteria displayed sensitivity to the antibiotics used in the study. Among G^−^ bacteria, *C. freundii* exhibited the highest sensitivity (10.33 mm), followed by *E. coli* (9.33 mm) and *S. marcescens* (8.67 mm). Notably, *E. coli* were the G^−^ bacteria that were most sensitive to antibiotics (28.67 mm). Additionally, IVEO demonstrated antimicrobial activity against the biofilm-forming bacteria *S. enterica*, with a zone of inhibition measuring 6.67 mm.

Using the broth microdilution method, the MIC_50_ and MIC_90_ were computed. The antimicrobial activity with the best MIC_50_ values (3.20 resp. 12.30 mg/mL) and MIC_90_ values (3.81 resp. 14.15 mg/mL) were observed against *E. faecalis* and *S. pneumoniae* from the tested G^+^ species. For *E. coli* and *C. freundii*, the mean MIC_50_ (1.55 resp. 6.41 mg/mL) and MIC_90_ (1.68 resp. 6.91 mg/mL) were determined as the lowest, compared to the other G^−^ species tested. The MBIC_50_ and MBIC_90_ values for *S. enterica* biofilm were 1.55 mg/mL and 1.72 mg/mL. The results obtained for the minimal inhibitory and minimal biofilm doses are shown in Table 3.

### 3.3. In Situ Antimicrobial Effect

**Figure 1:**
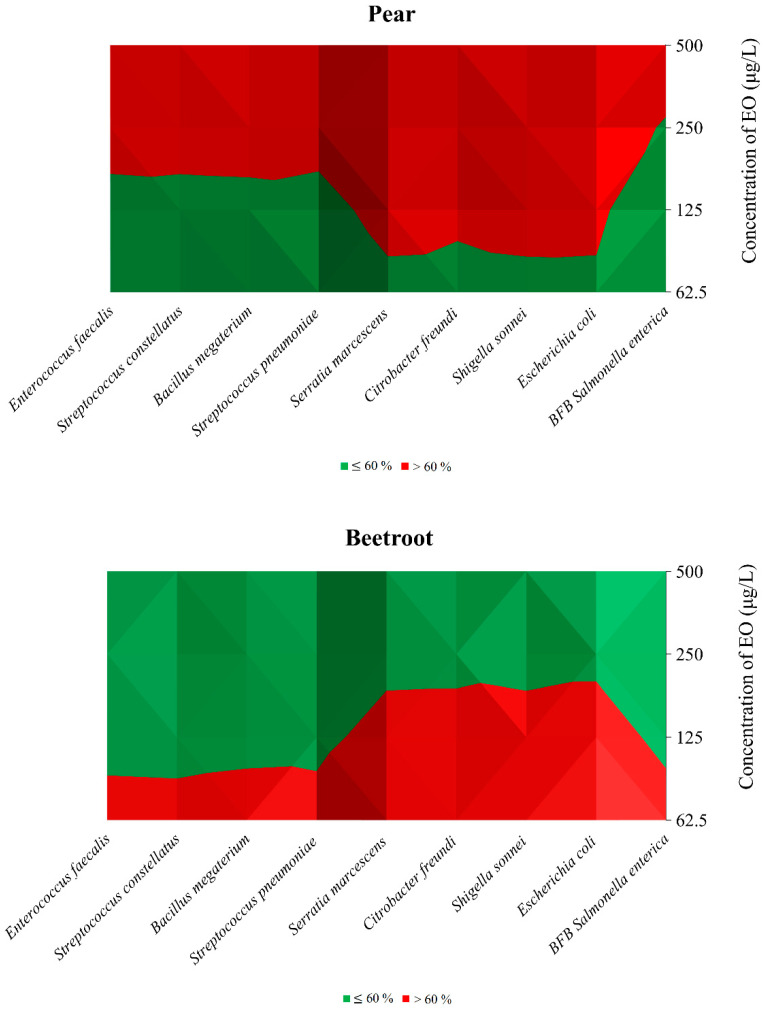
Isometric elaboration of the data in Table 4 (*In situ* analysis of the antimicrobial activity of the vapor phase of IVEO in pear and beetroot models). Green: ≤60%; Red: >60%.

To comprehensively assess the antibacterial properties of the IVEO, an *in situ* antimicrobial analysis was conducted using pear and beetroot as food models. The bacterial strains employed to determine the MIC_50_ and MIC_90_ values were also used for this evaluation. The outcomes of this assessment are presented in Table 4 and Figure 1. When IVEO was used to assess the development of *E. faecalis* on pear *in situ*, the results indicated that the concentration of 500 μg/L had the highest inhibitory effect (77.29%). The highest tested IVEO concentration (76.68 vs. 76.22%) substantially reduced the growth of *S. pneumoniae* and *S. constellatus* in pears. The highest percentage of 75.21% IVEO inhibition against *P. megaterium* was observed at an applied concentration of 500 μg/L. At the highest dose examined (74.64%), IVEO demonstrated significant antibacterial activity against G^−^ bacterial species, specifically *E. coli*. The best growth-suppressing effect towards biofilm-producing bacteria *S. enterica* IVEO showed in the highest concentration (77.06%) on the pear models. It was frequently noted that IVEO had a moderately suppressive effect when the growth of G^+^ biofilm-forming *S. enterica* bacteria on beets was assessed *in situ*. Moreover, the findings demonstrated that IVEO showed the highest efficacy in inhibiting the growth of G^−^ bacterial strains towards *S. sonnei* and *C. freundii* growing on beetroot in a treatment with the lowest dose applied (76.95% vs. 76.73%).

In conclusion, as shown in Figure 1, when the IVEO was applied to pear models with concentrations of 500 μg/L, all tested microorganisms were inhibited (>60%). This percentage was maintained, except for BFB *Salmonella enterica*, even at concentrations of 250 μg/L of IVEO.

Contrastingly, in the case of beetroot models, a strong inhibitory effect (>60%) is observed only at concentrations of 62.5 μg/L of IVEO for all of the tested microorganisms. Subsequently, the effect tends to decrease as the concentration increases, with a strong impact confirmed only for Gram-negative bacteria at a concentration of 125 μg/L (Figure 1).

### 3.4. Microbiological Analyses of Pumpkin Prepared by Sous Vide Method

**Figure 2:**
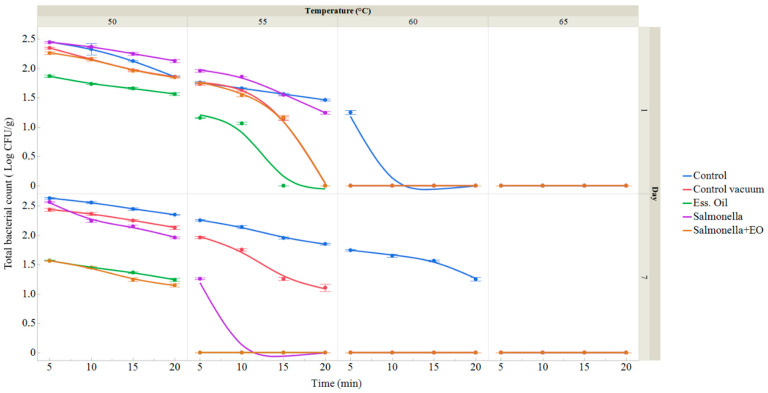
Total viable count of pumpkin sous vide samples on days 1 and 7. The data represent the mean (±SD) of three samples. The control group denotes a fresh pumpkin sample treated at 50–65 °C for 5 to 25 min after being packed in polyethylene bags and stored at 4 °C. The control vacuum group represents a fresh pumpkin sample treated under the same conditions but vacuum-packed in polyethylene bags and kept at 4 °C. The EO group comprises vacuum-packed fresh pumpkin treated with 1% IVEO, stored at 4 °C, and treated for 5–25 min at 50–65 °C. The *Salmonella* group includes vacuum-packed fresh pumpkin treated with *S. enterica*, stored at 4 °C, and treated for 5–25 min at 50–65 °C. The *Salmonella* + EO group consists of vacuum-packed fresh pumpkin treated with both *S. enterica* and 1% IVEO, stored at 4 °C, and treated for 5–25 min at 50–65 °C.

Raw pumpkin samples underwent microbiological analysis to confirm the presence of *S. enterica* on XLD agar, where typical colonies were confirmed. On day 0, the total bacterial count (TBC) was 2.21 ± 0.04 log CFU/g, and no coliform bacteria were identified. The microbiological quality of the vacuum-packed pumpkins was evaluated based on TBC on the first and seventh day of storage (Figure 2). The total bacterial counts in the control group ranged from 1.25 ± 0.04 in the group treated with 60 °C for 5 min to 2.45 ± 0.02 log CFU/g in the group treated with 50 °C for 5 min on day 1 and from 1.25 ± 0.03 in group treated with 60 °C to 2.63 ± 0.02 log CFU/g in the group treated with 50 °C for 5 min on day 7. Within the vacuum-packed group, the TBC varied between 1.14 ± 0.03 in the group treated at 55 °C for 15 min and 2.35 ± 0.02 log CFU/g in the group treated at 50 °C for 5 min on day 1 and between 1.11 ± 0.06 treated at 55 °C for 20 min and 2.44 ± 0.03 log CFU/g in the group treated at 50 °C for 5 min on day 7. In the vacuum-packed and IVEO-treated groups, the TCB log CFU/g ranged from 1.06 ± 0.02 in the group treated at 55 °C for 10 min to 1.87 ± 0.02 in the group treated at 50 °C for 5 min on day 1 and from 1.24 ± 0.03 in the group treated at 50 °C for 20 min to 1.57 ± 0.01 log CFU/g in the group treated at 50 °C for 5 min on day 7. For the group with *S. enterica*, the TBC ranged from 1.24 ± 0.02 in the group treated at 55 °C for 20 min to 2.45 ± 0.02 log CFU/g in the group treated at 50 °C for 5 min on day 1 and from 1.26 ± 0.02 in the group treated at 55 °C for 5 min to 2.57 ± 0.01 log CFU/g in the group treated at 50 °C for 5 min on day 7. In the group with vacuum-packing, IVEO treatment and presence of *S. enterica*, the TBC ranged from 1.17 ± 0.03 log CFU/g in group treated at 55 °C for 15 min to 2.25 ± 0.02 log CFU/g in the group treated at 50 °C for 5 min on day 1 and from 1.22 ± 0.02 in the group treated at 50 °C for 20 min to 2.26 ± 0.02 log CFU/g in the group treated with 50 °C for 5 min on day 7. Overall, the groups treated with IVEO and *S. enterica* exhibited lower bacterial counts. Figure 2 illustrates the results of the total bacterial count (TBC) (expressed in log CFU/g) on the first and seventh days, with treatments conducted at temperatures ranging from 50 to 65 °C for durations of 5 to 20 min.

**Figure 3:**
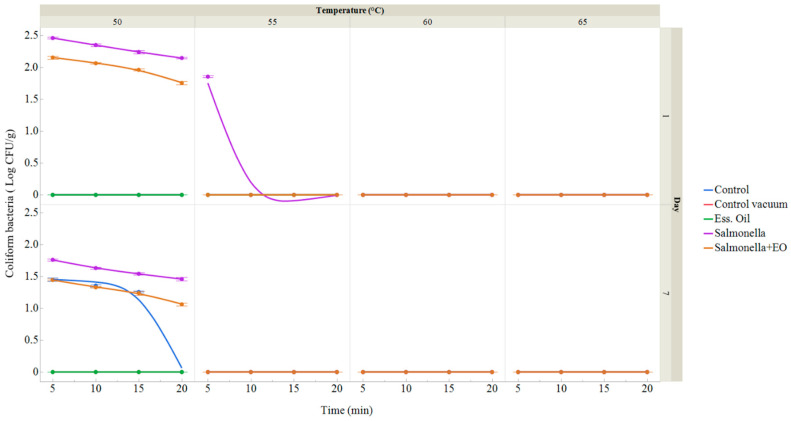
Coliform bacteria on pumpkin sous vide samples on days 1 and 7. The data represent the mean (±SD) of three samples. The control group denotes a fresh pumpkin sample treated at 50–65 °C for 5 to 25 min after being packed in polyethylene bags and stored at 4 °C. The control vacuum group represents a fresh pumpkin sample treated under the same conditions but vacuum-packed in polyethylene bags and kept at 4 °C. The EO group comprises vacuum-packed fresh pumpkin treated with 1% IVEO, stored at 4 °C, and treated for 5–25 min at 50–65 °C. The *Salmonella* group includes vacuum-packed fresh pumpkin treated with *S. enterica*, stored at 4 °C, and treated for 5–25 min at 50–65 °C. The *Salmonella* + EO group consists of vacuum-packed fresh pumpkin treated with both *S. enterica* and 1% IVEO, stored at 4 °C, and treated for 5–25 min at 50–65 °C.

On the first day, no counts of coliform bacteria (CB) were performed in the IVEO group, vacuum-packed control group, or control group (Figure 3). The number of coliform bacteria in the control group ranged from 1.26 ± 0.02 in the group treated at 50 °C for 15 min to 1.45 ± 0.03 log CFU/g in the group treated at 50 °C for 5 min on the seventh day. After seven days, coliform bacteria were found neither in the group that used IVEO nor in the vacuum-packaged group. In the group to which *S. enterica* was added on the first day, the amount of CB varied from 1.86 ± 0.02 in the group treated at 55 °C for 5 min to 2.46 ± 0.02 log CFU/g in the group treated at 50 °C for 5 min, and on the seventh day the amount of CB ranged from 1.46 ± 0.03 in the group treated at 55 °C for 5 min to 1.76 ± 0.02 log CFU/g in the group treated at 50 °C for 5 min. The amount of CB in the group with the application of *S. enterica* ranged between 1.76 ± 0.03 in the group treated at 50 °C for 20 min and 2.16 ± 0.02 log CFU/g in the group treated at 50 °C for 5 min on the first day, and between 1.06 ± 0.02 in the group treated at 50 °C for 20 min and 1.45 ± 0.02 log CFU/g with treatment at 50 °C for 5 min on the seventh day within the treatment group provided with both IVEO and *S. enterica*. Figure 3 shows the results of the amount of coliform bacteria in VRBL (expressed in log CFU/g) on the first and seventh days, with treatments applied at temperatures ranging from 50 to 65 °C for durations of 5 to 20 min.

**Figure 4:**
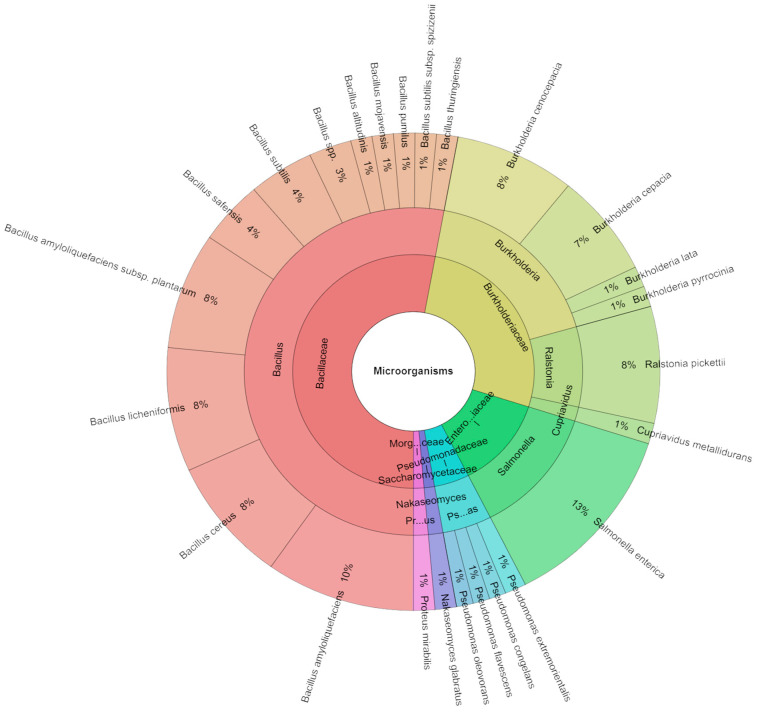
Krona chart: Species of microorganisms isolated from sous vide pumpkin after 1 day.

A total of 356 isolates were identified by mass spectrometry (Figure 4). A total of six families, eight genera, and twenty-five species were isolated from the vacuum-packed pumpkin samples on the first day of monitoring. The most isolated species was *S. enterica* (13%), followed *Bacillus amyloliquefaciens* (10%), *B. cereus*, and *B. licheniformis* (8%).

**Figure 5:**
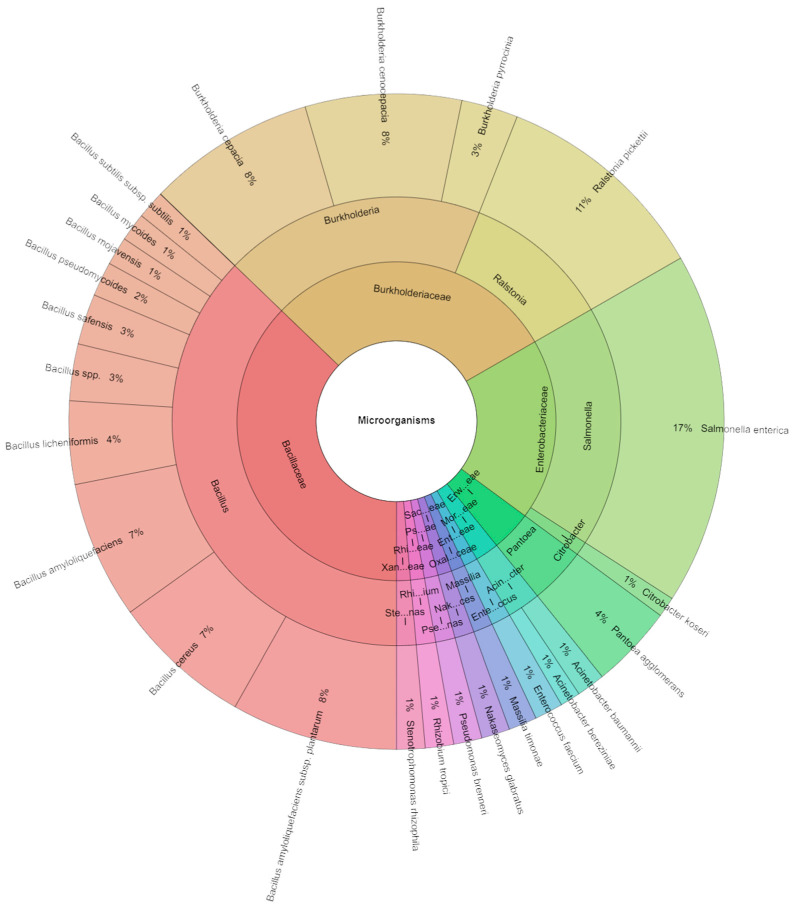
Krona chart: Species of microorganisms isolated from sous vide pumpkin after 7 days.

Mass spectrometry was used to identify 363 isolates in total (Figure 5), which were separated on the first day of observation from the sous vide pumpkin samples, comprising 11 families, 13 genera, and 25 species. *S. enterica* was the most isolated species (17%), followed by *Ralstonia picketii* (11%), *Bacillus amyloliquefaciens* subsp. *plantarum*, and *Burkholderia cepacia* (8%).

### 3.5. Antibiofilm Effect Evaluated by MALDI-TOF MS Biotyper

**Figure 6:**
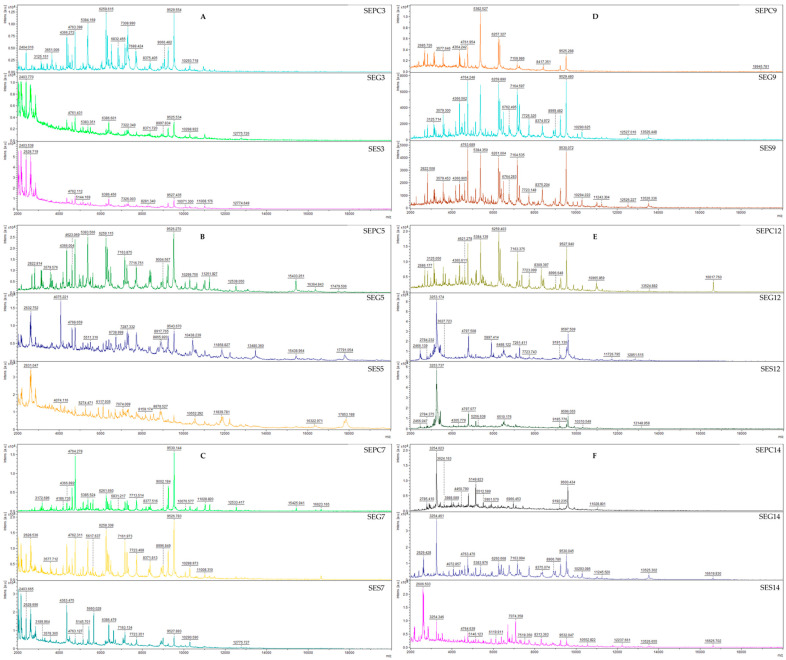
MALDI-TOF mass spectra of *S. enterica* on the third, fifth, seventh, ninth, twelve, and fourteenth days. **A**-day 3, **B**-day 5, **C**-day 7, **D**-day 9, **E**-day 12, **F**-day 14. S = stainless steel; G = glass; PC = planktonic cells; SE = *S. enterica*.

The antibiofilm efficacy of IVEO against *S. enterica*, a biofilm-producing bacteria, was assessed utilizing MALDI-TOF MS Biotyper mass spectrometry (Figure 6A–F). The untreated IVEO samples, which were used to generate the spectrum for the control group, consisted of planktonic cells and the biofilm was removed from the model surfaces. The planktonic cell control spectrum and the spectrum derived from the model surfaces exhibited analogous evolution. For the comparative analysis of molecular alterations between biofilm and the experimental group, only the control planktonic spectrum was used. MALDI-TOF MS Biotyper mass spectrometry was employed to investigate the impact of IVEO on biofilm-forming *S. enterica*. Comparisons of molecular changes in the biofilm were carried out using spectra from control groups—planktonic cells and untreated EO—demonstrating similar developments. At early biofilm stages (Figure 6A,B), the spectra were not in parallel with the planktonic spectra on days 3 or 5, indicating distinct protein profiles between control and experimental groups in young biofilms. The progressive spectra evolution suggested significant changes in the biofilm protein profile. The peaks in the experimental group on days 7 and 9 displayed an increased intensity compared to planktonic spectra, signifying altered protein profiles. Notably, on days 12 and 14, discernible effects of the IVEO on plastic and stainless-steel surfaces were observed (Figure 6E,F). These distinct modifications in the biofilm’s protein composition, particularly on the stainless-steel surface, appear to have influenced the bacterial biofilm’s homeostasis, contributing to its suppression.

**Figure 7:**
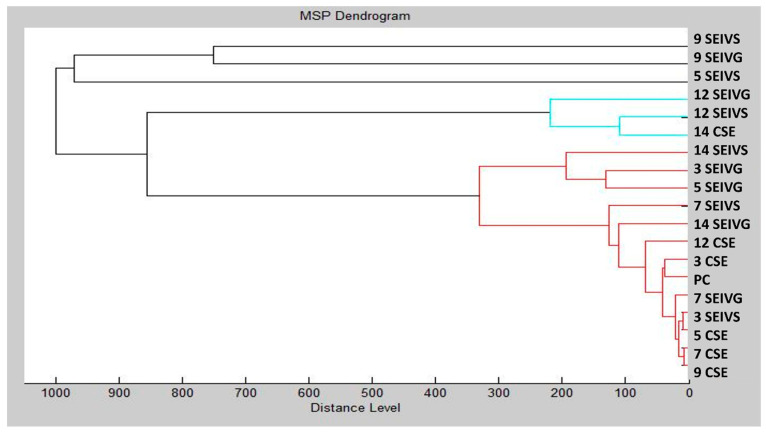
*S. enterica* dendrogram created with planktonic cell MSPs and control. S = stainless steel; G = glass; PC = planktonic cells; SE = *S. enterica*.

A dendrogram, constructed based on MSP distances, was used to visually represent the structural similarities within the biofilm. The resulting dendrogram (Figure 7) illustrated that the initial biofilm and control phases (three SEIVS and five CSE) and later biofilm phases (seven CSE and nine CSE) demonstrated the shortest MSP distances, closely resembling the control and planktonic spectra. Short MSP distances between control groups indicated analogous protein profiles. On day 3, the early biofilm forms of planktonic cells exhibited comparably short MSP distances to the control, as revealed by the mass spectra analysis. The study highlighted a gradual increase in MSP distance within the experimental group over time, reaching its peak on day 9. These findings provide insights into how IVEO influences the homeostasis of the *S. enterica* biofilm.

### 3.6. Insecticidal Activity of IVEO

Table 5 shows the results of the evaluation of the insecticidal efficacy of the IVEO against *H. axyridis*. The data indicate that the highest insecticidal activity was observed at applied doses of 50% and 100% of the tested EO. However, when administered at doses of 6.25% and 3.125%, the IVEO did not demonstrate significant repellent qualities against *H. axyridis*. Notably, a concentration of 12.5% exhibited an impact on the *H. axyridis* population while a concentration of 25% was effective against 60% and 75% of the insects, respectively.

## 4. Discussion

The GC/MS analyses indicated that (*E*)-anethole constitutes over 90% of the total composition of the tested essential oil. Our findings align with the existing literature, which consistently identifies (*E*)-anethole as the predominant component, typically comprising between 70% and 94% of the oil’s composition [34,35,36,37]. Besides this phenylpropanoid, estragole was detected with a percentage value of 4.8%. Additionally, the IVEO sample was characterized by small amounts (total of 4.0%) of monoterpene compounds, aromatic compounds, and sesquiterpene hydrocarbons. In agreement with the data in the literature, other components such as α-pinene, limonene, 1,8-cineole, *p*-anis aldehyde, δ-3-carene, α-(*E*)-bergamotene were detected in smaller quantities [36,37]. The quantitative differences in these volatile compounds can be ascribed to various factors, including the timing of harvest, the geographical origin of the plant, and whether the plant material utilized for distillation is fresh or dried [35]. The previous literature reports indicated (*E*)-anethole as the major secondary metabolite responsible for the antimicrobial effectiveness of *Foeniculum vulgare* var. *azoricum* (Mill.) Thell. [38]. Bearing that in mind, our study was further designed to investigate the different levels of the antimicrobial activity of IVEO.

Thus, we initially confirmed the antibacterial efficacy of star anise EO against *S. enterica* biofilm-forming, G^+^, and G^−^ bacteria using both the disk diffusion and microdilution methods. It is commonly observed that G^+^ bacteria are more susceptible to the effects of EOs than G^−^ bacteria [39,40], which was not confirmed in our study. Based on the results obtained here, we can conclude that the bacteria that were most sensitive to the effects of the IVEO were G^−^ *E. coli*. Similar to our results, Freire et al. [41] found that IVEO was most effective against *E. coli* strains. However, Noumi et al. also showed the better efficiency of IVEO towards Gram-positive bacteria (*Staphylococcus aureus*) compared to Gram-negative bacteria (*Pseudomonas aeruginosa*, *S. flexeneri*, and *Vibrio vulnificus*) [42]. Additionally, in the antimicrobial assessment of IVEO using the disc paper method, this oil showed a better effectiveness against *S. aureus* compared to *E. coli* [43]. Moreover, previously conducted studies indicated that IVEO exhibits a significant efficacy against G^+^ bacteria, such as methicillin-resistant *Staphylococcus aureus* (MRSA) [34]. The authors propose that this EO could serve as a valuable tool in addressing resistant strains and may offer a novel approach to combating microorganisms resistant to multiple drugs. Furthermore, a qualitative evaluation of the susceptibility testing potential of IVEO was conducted using a collection of multidrug-resistant (MDR) clinical isolates, encompassing *S. pneumoniae*, *S. aureus*, *K. pneumoniae*, *E. coli*, *A. baumannii*, and *P. aeruginosa*. The study’s results not only confirmed the heightened efficacy of IVEO against G^+^ strains but also demonstrated its potent antibacterial activity against each MDR clinical isolate tested [44]. In an independent investigation, the antibacterial properties of this essential oil were evaluated against six distinct pathogens, of which *Staphylococcus aureus* was the most susceptible, followed by *Staphylococcus epidermidis* and *Enterobacter cloacae* [45]. Additionally, it was noted that essential oils made from star anise were inactive against *Salmonella typhi* but had a potent activity against *S. aureus* and *E. coli* [46]. The variability in the results can be ascribed to a multitude of factors, including the utilization of diverse bacterial strains in antimicrobial assessments, the distribution of active compounds within the EO, and their potential additive, antagonistic, or synergistic effects. Several studies have highlighted that the use of crude plant EOs, owing to their high concentration of active compounds, exhibits biological activity across a broad spectrum of inhibitory zone diameters [47]. When trans-anethole was tested against test fungi, its inhibitory activity was found to be comparable to that of IVEO, with IC_50_ values that were close to those of the oil. This finding suggested that trans-anethole played a significant role in the antifungal characteristics of IVEO [21]. At lower concentrations of 100 ppm, the star anise essential oil exhibited strong antifungal action and completely inhibited (100%) the development of *F. graminearum*, *F. solani*, and *F. oxysporum*. Nevertheless, at 200 ppm, *F. verticillioides* was totally suppressed [48].

Considering the promising results obtained from standard methods such as disc diffusion and microdilution assays, which demonstrated the considerable antimicrobial potential of IVEO in direct contact applications, the subsequent objective of this study was to assess its efficacy in the vapor phase. The findings revealed that the vapor phase of IVEO exhibited a significant effectiveness in inhibiting the growth of *E. coli* at a higher concentration in the pears model and against *S. sonnei* at a lower concentration in the beetroot model. In Čmiková et al.’s [49] investigation, the antibacterial potential of the IVEO vapor phase was evaluated against the growth of G^+^, G^−^ bacteria, and yeasts on carrots. The results indicated that IVEO is highly effective against both G^+^ and G^−^ bacteria similar to those in our study. However, the best antibacterial effect that the IVEO showed in the highest applied concentration (500 μL/L) was against the G^+^ strains *M. luteus* (95.87 ± 4.58%) and *S. aureus* (95.64 ± 3.26%). The data from the literature also suggest the possible use of IVEO and its main component (trans-anethole) as fumigants in the prevention of post-harvest plant diseases caused by *Pythium aphanidermatum* and *Botryodiplodia theobromae* [21]. These results imply the possible use of the IVEO in protecting fruits and vegetables against post-harvest infections.

Our study’s subsequent objective was to assess the microbiological integrity of pumpkin prepared sous vide after the application of IVEO inoculated with *S. enterica* bacteria. Overall, the findings revealed the notable antimicrobial effects of IVEO against *S. enterica* in the pumpkin model. The predominant species isolated from the group treated with this bacterium was *S. enterica*. Additionally, the species *R. picketii*, *B. amyloliquefaciens*, *B. cereus*, *B. licheniformis*, and others have been isolated from the natural microbiome. Our study aimed to provide a ready-to-use product with an extended shelf life, unchanged nutritional content, and microbiological quality while minimizing customer labor. For this reason, preservation methods such as vacuum packing and low-temperature storage, rather than high-temperature heating like the sous vide method, and vacuum-sealed foods, are employed in conjunction with EOs to prolong the shelf life and prevent the decline in the microbial quality of minimally processed foods. Coliform, *S. aureus*, *E. coli*, and *Lactobacillus* species were not found in samples of vacuum- or non-vacuum-packed pumpkin pieces stored for 15 and 20 days [50]. The study documented mold and yeast counts ranging from 2.19 to 2.41 log CFU/g and aerobic bacteria counts ranging from 2.76 to 4.44 log CFU/g. These counts surpassed those observed in our investigation involving sous vide-treated pumpkin. Total bacterial counts in vegetables serve as indicators of microbial load, although they do not inherently convey the impact, whether positive or negative, of the population [51]. The counts provide an indication of the product’s quality. In a study by Roura et al. [52], minimally processed pumpkin pieces stored at 10 to 12 °C for 15 days showed mesophilic aerobic bacteria populations of 8.50 log CFU/g (3.50 × 10^8^ CFU/g). Sasaki et al. [53] observed the counts of aerobic bacteria in diced pumpkins frozen at 5 °C (0.60 log CFU/g on day 0, 5.50 log CFU/g on day 6, and 6.90 log CFU/g on day 12). Baskaran et al. [54] found that soaking diced pumpkins in a 0.2% citric acid and 0.1% potassium metabisulfite solution resulted in aerobic bacteria counts of 5.50 log CFU/g (32.40 × 10^4^ CFU/g) on day 25 at 5 °C. In partially processed pumpkins wrapped in polyethylene and stored at 10 to 12 °C in plastic containers, Roura et al. [52] reported mold and yeast counts of 6.80 log CFU/g and 6.30 × 10^6^ CFU/g onat day 15 [55]. Moreover, it has been demonstrated that essential oils are useful in inhibiting the growth and decreasing the quantity of foodborne pathogens, including *S. dysenteriae*, *L. monocytogenes*, *B. subtilis*, *S. typhimurium*, *Salmonella* spp., and *E. coli* O157:H7 [56,57]. There is growing interest in the utilization of EOs as potent agents to combat antimicrobial contamination in minimally processed food [58]. The essential oils possess strong antibacterial properties [59]. The antibacterial activity of EOs in vegetable meals is positively impacted by low storage temperatures and reduced pH levels [60]. Researchers conducted several trials utilizing EOs to develop vegetable packaging, which showed promising antibacterial results [61,62,63,64]. Numerous studies have been conducted on the use of essential oils in antimicrobial packaging to increase the stability and shelf life of vegetables [65,66,67,68]. An analysis of communities associated with pumpkins revealed that *Pseudomonas* and *Bacillus* are both major inhabitants and antagonists of plants [69,70,71]. It has been determined that *Pseudomonas putida*, *Pseudomonas syringae*, *P. viridiflava*, and *Pseudomonas fluorescens* are all different entities. In several pumpkin microhabitats, the following bacteria were found: *Bacillus*, *B. weihenstephanensis*, *B. flexus*, *B. psychrodurans*, *B. siralis*, *B. indicus*, *B. subtilis*, *B. gibsonii* [72]. Some of these species were found in the sous vide pumpkin in our study and were identified with mass spectrometry.

Given the significant inhibitory effects of IVEO against *S. enterica* inoculated on pumpkin, the subsequent objective of this study was to explore its potential in suppressing the biofilm formation of this bacterium. An initial crystal violet assay unveiled the remarkable potency of this essential oil in inhibiting biofilm formation, prompting further investigations. Through the utilization of MALDI-TOF analysis, we demonstrated the capability of IVEO to modify the protein profile of biofilms formed on plastic and stainless-steel surfaces from the early stages of the experiment. Like the results obtained herein, Luís et al. [73] demonstrated the ability of IVEO to inhibit quorum sensing, eradicate the already formed biofilm, and disperse pre-formed biofilms of *A. baumanii*. Another investigation also found IVEO to be effective in eradicating *S. aureus* biofilm-forming bacteria on polystyrene and glass surfaces [42]. In the same study, the authors discovered that IVEO exhibited a stronger inhibitory effect on the migration of *Pseudomonas aeruginosa* PAO1, with a 38% inhibition rate at a concentration of 100 µg/mL, when compared to its primary component, trans-anethole [42].

In our investigation, IVEO showed insecticidal effects against *H. axyridis* at higher concentrations with high mortality results. Also, the EO of *I. verum* showed insecticidal activity in a study by Freitas et al. [74]. *I. verum* fruits have previously been shown to have insecticidal properties [75,76,77]. As reported by Gomes da Rocha Voris et al. [78] and Popović et al. [79], the EO derived from this plant demonstrates insecticidal effects against *Aedes aegypti* larvae and adults, as well as against the insect *Tribolium confusum*, thereby contributing to the preservation of grains. The environmental mites *Dermatophagoides pteronyssinus* [80] and the tick nymph *Ixodes ricinus* [81] have also been reported to have acaricide activity. In addition to the findings of this investigation, star anise essential oil shows promise in the fight against *H. axyridis*.

## 5. Conclusions

Bearing in mind the growing demand in the food industry for novel food preservatives from natural sources, this study was designed to evaluate the effectiveness of IVEO as antimicrobial agent. The GC/MS study identified (*E*)-anethole, which is already a recognized antimicrobial, as the major constituent of the investigated EO. The antimicrobial activity testing revealed strong antibacterial effects, particularly against *E. coli*, both *in vitro* and *in situ* in vegetable and fruit models. These promising results suggest the potential future application of IVEO to extend the shelf life of food products and its suitability for preserving pears and beetroots. Sous vide technology is a low-temperature, long-term, vacuum-packed-food cooking method. Thanks to this application, the distinct flavor and nutritional value of the food are preserved even if it is cooked and vacuum-packed. In addition, through this technology, products with excellent nutritional content, good quality, and a long shelf life are possible. Even though it has more benefits than conventional cooking techniques, harmful microorganisms might proliferate and endanger human health if the temperature is too low or the cooking period is too short. Because of this, it is crucial to follow temperature–time guidelines. In this study, it was found that utilizing IVEO in conjunction with sous vide technology prevented the growth of *S. enterica* in pumpkin. Moreover, we have demonstrated notable antimicrobial and antibiofilm effects of IVEO against biofilm-forming *S. enterica*, as evidenced on various surfaces using the crystal violet method and the MALDI-TOF MS Biotyper. Additionally, our investigation showed that IVEO exhibited insecticidal effects against the Asian lady beetle.

## Figures and Tables

**Table 1:** Volatile constituents of IVEO.

No	RI (Lit.) ^a^	RI (Calc.) ^b^	Compound ^c^	% ^d^
Monoterpenes				2.0
Monoterpene hydrocarbons				1.8
1	939	934	α-pinene	0.7
2	1002	1006	α-phellandrene	0.4
3	1011	1008	δ-3-carene	0.2
4	1026	1027	*o*-cymene	Tr ^e^
5	1029	1032	limonene	0.5
6	1059	1063	γ-terpinene	tr
Oxygenated monoterpenes				0.2
Monoterpene epoxides				0.2
7	1031	1035	1,8-cineole	0.2
Phenylpropanoids				93.2
8	1196	1198	estragole	4.8
9	1284	1290	(*E*)-anethole	88.4
Aromatic compounds				0.9
10	1244	1250	anisole	0.3
11	1250	1255	*p*-anis aldehyde	0.6
Sesquiterpenes				1.1
sesquiterpene hydrocarbons				1.1
12	1375	1377	α-ylangene	0.1
13	1419	1419	(*E*)-caryophyllene	0.4
14	1434	1434	α-(*E*)-bergamoten	0.5
15	1505	1506	β-bisabolene	0.1
Total				97.2

^a^ Literature values of retention indices on HP-5MS column; ^b^ calculated values of retention indices on HP-5MS column; ^c^ identified compounds; ^d^ percentage amounts of identified compounds; ^e^ tr-compounds detected in amounts less than 0.1%.

**Table 2:** Disc diffusion method antimicrobial activity in mm.

Microorganism	Inhibition Zone	ATB
Gram-positive bacteria		
*Enterococcus faecalis* CCM4224	7.33 ± 0.58 ^bc^	27.67 ± 0.58 ^bc^
*Streptococcus constellatus* CCM 4043	5.33 ± 0.57 ^d^	28.33 ± 0.58 ^abc^
*Priestia megaterium* CCM 2007	5.67 ± 0.58 ^cd^	29.33 ± 0.58 ^a^
*Streptococcus pneumoniae* CCM 4501	6.33 ± 1.15 ^cd^	27.67 ± 0.57 ^bc^
Gram-negative bacteria		
*Serratia marcescens* CCM 8587	8.67 ± 0.58 ^ab^	27.00 ± 0.05 ^c^
*Citrobacter freundii* CCM 7187	10.33 ± 0.58 ^a^	27.67 ± 0.58 ^bc^
*Shigella sonnei* CCM 4421	7.33 ± 0.59 ^bc^	28.00 ± 0.58 ^abc^
*Escherichia coli* CCM 3954	9.33 ± 0.58 ^a^	28.67 ± 0.58 ^ab^
Biofilm-forming bacteria (BFB)		
*Salmonella enterica*	6.67 ± 0.58 ^cd^	28.33 ± 0.58 ^abc^

Data are the mean (±SD) of 3 samples. Different letters in each column refer to significant differences (Tukey, *p* < 0.05). ATB = Antibiotics (G^−^ cefoxitin and G^+^ gentamicin 30 µg/disc).

**Table 3:** Minimal inhibition concentration and minimal biofilm inhibition concentration of IVEO in mg/mL.

Microorganism	MIC_50_	MIC_90_
Gram-positive bacteria		
*Enterococcus faecalis* CCM4224	3.20 ± 0.03 ^e^	3.81 ± 0.16 ^de^
*Streptococcus constellatus* CCM 4043	43.41 ± 1.46 ^a^	46.72 ± 1.12 ^a^
*Priestia megaterium* CCM 2007	23.27 ± 1.51 ^b^	26.71 ± 0.99 ^b^
*Streptococcus pneumoniae* CCM 4501	12.30 ± 0.45 ^c^	14.15 ± 0.57 ^c^
Gram-negative bacteria		
*Serratia marcescens* CCM 8587	12.57 ± 0.84 ^c^	14.60 ± 2.82 ^c^
*Citrobacter freundii* CCM 7187	6.41 ± 0.16 ^d^	6.91 ± 0.13 ^d^
*Shigella sonnei* CCM 4421	12.27 ± 0.24 ^c^	12.61 ± 0.23 ^c^
*Escherichia coli* CCM 3954	1.55 ± 0.01 ^e^	1.68 ± 0.10 ^e^
Biofilm-forming bacteria		
*Salmonella enterica*	1.51 ± 0.05 ^e^	1.72 ± 0.14 ^e^

Data are the mean (±SD) of 3 samples. Different letters in each column refer to significant differences (Tukey, *p* < 0.05). MIC_50_ and MIC_90_ values are in mg/mL.

**Table 4:** *In situ* analysis of the antimicrobial activity (in %) of IVEO in the vapor phase on pear and beetroot.

Food Model	Microorganisms	Concentration of EO in μg/L			
		62.5	125	250	500
**Pear**					
	Gram-positive bacteria				
	*Enterococcus faecalis*	45.27 ± 1.56 ^b^	55.11 ± 1.53 ^b^	66.34 ± 1.39 ^b^	77.29 ± 1.10 ^b^
	*Streptococcus constellatus*	43.70 ± 1.21 ^b^	55.76 ± 2.10 ^b^	65.55 ± 1.15 ^b^	76.22 ± 2.07 ^b^
	*Priestia megaterium*	44.30 ± 2.21 ^b^	56.07 ± 2.24 ^b^	66.03 ± 1.63 ^b^	75.21 ± 2.17 ^b^
	*Streptococcus pneumoniae*	44.81 ± 1.76 ^b^	53.89 ± 0.74 ^b^	66.99 ± 1.33 ^b^	76.68 ± 1.21 ^b^
	Gram-negative bacteria				
	*Serratia marcescens*	55.41 ± 1.65 ^a^	65.99 ± 1.80 ^a^	76.48 ± 1.65 ^a^	86.74 ± 1.89 ^a^
	*Citrobacter freundii*	54.92 ± 1.74 ^a^	63.12 ± 1.63 ^a^	74.81 ± 1.18 ^a^	86.80 ± 1.70 ^a^
	*Shigella sonnei*	55.81 ± 1.70 ^a^	65.52 ± 1.94 ^a^	76.22 ± 2.62 ^a^	85.92 ± 2.18 ^a^
	*Escherichia coli*	55.30 ± 1.30 ^a^	65.76 ± 1.85 ^a^	75.22 ± 2.18 ^a^	87.07 ± 2.27 ^a^
	Biofilm-forming bacteria				
	*Salmonella enterica*	15.45 ± 2.63 ^c^	36.44 ± 1.74 ^c^	57.54 ± 2.57 ^c^	77.06 ± 1.72 ^b^
**Beetroot**					
	Gram-positive bacteria				
	*Enterococcus faecalis*	65.73 ± 2.16 ^b^	55.11 ± 1.53 ^b^	45.44 ± 1.53 ^b^	35.33 ± 2.73 ^b^
	*Streptococcus constellatus*	65.33 ± 2.26 ^b^	54.71 ± 1.00 ^b^	43.70 ± 0.95 ^b^	34.74 ± 1.06 ^b^
	*Priestia megaterium*	66.96 ± 2.29 ^b^	55.77 ± 0.96 ^b^	45.47 ± 0.63 ^b^	35.77 ± 2.04 ^b^
	*Streptococcus pneumoniae*	65.40 ± 2.19 ^b^	56.25 ± 1.53 ^b^	45.17 ± 1.54 ^b^	34.97 ± 2.74 ^b^
	Gram-negative bacteria				
	*Serratia marcescens*	76.55 ± 1.94 ^a^	65.33 ± 2.28 ^a^	55.85 ± 1.01 ^a^	45.77 ± 2.05 ^a^
	*Citrobacter freundii*	76.73 ± 2.28 ^a^	65.37 ± 2.22 ^a^	56.22 ± 1.57 ^a^	44.74 ± 0.95 ^a^
	*Shigella sonnei*	76.95 ± 2.39 ^a^	67.09 ± 2.35 ^a^	54.44 ± 1.52 ^a^	45.77 ± 1.83 ^a^
	*Escherichia coli*	75.44 ± 1.27 ^a^	67.25 ± 1.62 ^a^	56.47 ± 2.22 ^a^	44.44 ± 1.49 ^a^
	Biofilm-forming bacteria				
	*Salmonella enterica*	66.44 ± 2.28 ^b^	56.11 ± 2.19 ^b^	44.44 ± 1.07 ^b^	34.40 ± 2.61 ^b^

Data are the mean (±SD) of 3 samples. Different letters in each column (for each type: pear and beetroot) refer to significant differences (Tukey, *p* < 0.05).

**Table 5:** Insecticidal activity of IVEO against *Harmonia axyridis*.

Concentration (%)	Number of Living Individuals	Number of Dead Individuals	Insecticidal Activity (%)
100	0	100	100.00 ± 0.00
50	10	90	90.00 ± 0.00
25	25	75	75.00 ± 0.00
12.5	40	60	60.00 ± 0.00
6.25	50	50	50.00 ± 0.00
3.125	65	35	35.00 ± 0.00
Control group	100	0	0.00 ± 0.00

## Data Availability

The original contributions presented in the study are included in the article, further inquiries can be directed to the corresponding author.
